# Mitomycin C induces apoptosis in cultured corneal fibroblasts derived from type II granular corneal dystrophy corneas

**Published:** 2008-06-30

**Authors:** Tae-im Kim, Seung-il Choi, Hyung Keun Lee, Young Jae Cho, Eung Kweon Kim

**Affiliations:** 1 Corneal Dystrophy Research Institute, Department of Ophthalmology, Yonsei University, College of Medicine, Seoul, Korea; 2 BK21 Project Team of Nanobiomaterials for Cell-based Implants, Yonsei University, Seoul, Korea

## Abstract

**Purpose:**

The present study investigated the effect of mitomycin C (MMC) on cell viability, apoptosis, and transforming growth factor beta-induced protein (TGFBIp) expression in cultured normal corneal fibroblasts and heterozygote or homozygote granular corneal dystrophy type II (GCD II) corneal fibroblasts.

**Methods:**

Keratocytes were obtained from normal cornea or from heterozygote or homozygote GCD II patients after lamellar or penetrating keratoplasty. To measure cell viability, corneal fibroblasts were incubated with 0.02% MMC for 3 h, 6 h, and 24 h or with 0%, 0.01%, 0.02%, and 0.04% MMC for 24 h and then tested using lactate dehydrogenase (LDH) and 3-[4,5-demethylthiazol-2,5-diphenyl-2H-tetrazolium bromide] (MTT) assays. To measure apoptosis, cells were analyzed by FACS analysis and annexin V staining. *Bcl-xL*, *Bax*, and *TGFBI* mRNA expression was measured using reverse transcription polymerase chain reaction (RT–PCR) assays. Cellular and media levels of TGFBIp protein were measured by immunoblotting.

**Results:**

MTT and LDH assays showed that MMC reduced cell viability in all three cell types in a dose-dependent and time-dependent manner (p<0.05). FACS analysis and annexin V staining showed that MMC caused apoptosis with GCD II homozygote cells being most affected. RT–PCR analysis showed that MMC decreased *Bcl-xL* mRNA expression and increased *Bax* mRNA expression in all cell types. RT–PCR and immunoblotting analysis showed that MMC reduced *TGFBI* mRNA levels and cellular and media TGFBIp protein levels in all cell types.

**Conclusions:**

MMC induced apoptosis, and the effects of MMC were greatest in GCD II homozygote cells. MMC also reduced the production of TGFBIp in all three types of corneal fibroblasts. These findings may explain the additional therapeutic effect of MMC in GCD II patients.

## Introduction

Abnormal deposition of extracellular matrix proteins is a key event related to the loss of corneal transparency in corneal dystrophies. The extracellular transforming growth factor beta-induced protein gene (*TGFBI*), which is located on 5q31, has been studied in relation to corneal dystrophies and several mutations have been found [1-4]. The gene encodes a 683 amino acid protein, which has been given several names including keratoepithelin [5], βigh3 protein [6], and TGFBIp. Antibodies directed against TGFBIp were found to react to corneal deposits in *TGFBI*-related corneal dystrophies [7]. While the mechanism underlying pathological deposition of *TGFBI* remains to be fully understood, the main component of the deposit is TGFBIp derived from mutant *TGFBI*.

Granular corneal dystrophy type II (GCD II; Avellino corneal dystrophy) represents a mixed-type corneal dystrophy resulting from an R124H mutation of TGFBIp, which is activated by TGF-β [8,9]. GCD II is more common in Asia than GCD type I, which is associated with an R555W mutation [10,11].

While phototherapeutic keratectomy (PTK) is the recommended treatment for granular corneal dystrophy, a major problem has been recurrence after treatment [12]. Similarly, penetrating keratoplasty can be used to replace the opaque cornea, but deposits can recur along the suture tract and even on the grafted donor cornea within several years of surgery [13]. Recently, refractive surgery has become a more commonly performed procedure. As such, reportedly severe aggravation of corneal deposits after refractive surgical procedures has made GCD II a cause for concern [14].

Mitomycin C (MMC) is widely used in the ophthalmic field due to its antiproliferative activity, which results from an inhibition of DNA synthesis secondary to alkylation [15-17]. The use of MMC to prevent subepithelial opacity after refractive procedures has resulted in promising outcomes via apoptosis of keratocytes [18,19]. Keratocyte apoptosis is the earliest stromal change observable after epithelial injury and is considered an initiator of the corneal wound healing response. As such, the inhibition of keratocyte apoptosis could result in diminished keratocyte activation. Therefore, regulating keratocyte apoptosis may be an effective way to control the response to wounding [20].

We have previously reported that the intraoperative use of topical 0.02% MMC in conjunction with PTK prevented or delayed GCD II recurrence [21]. Despite the clinical use of MMC, there are few supporting in vitro studies that have investigated its mechanism of action. The present study examined the effect of MMC on cultured corneal fibroblasts derived from normal, heterozygote GCD II, and homozygote GCD II individuals. The study examined the effect of MMC on cell viability, apoptosis, and TGFBIp production.

## Methods

### Culture of corneal fibroblasts from normal, heterozygote granular corneal dystrophy type II, and homozygote granular corneal dystrophy type II patients

This study adhered to the tenets of the Declaration of Helsinki. Keratocyte primary cultures were prepared from corneas from the eye bank or from heterozygote or homozygote GCD II patients after penetrating or lamellar keratoplasty (Severance hospital IRB approved this study-IRB-2006–0139). GCD II diagnoses were based on the results of DNA analysis for the R124H *TGFBI* mutation. Stromal explants were prepared by first removing the epithelium and endothelium and then culturing the explants in 10% fetal bovine serum (FBS)/Dulbecco's Modified Eagle's medium (DMEM) at 37 °C in 5% CO_2_ and in six well tissue culture plates. Corneal fibroblasts migrated from the explants along the bottom of the plates. Cells were confluent within 15–21 days. Cells were harvested by enzymatic detachment using 0.05% trypsin at 37 °C for 3 min followed by centrifugation at 1400 rpm for 5 min and aspiration of the supernatant. The cell pellet was then resuspended in 20 ml of the medium and cultured in 75 ml flasks at 37 °C in 5% CO_2_ until confluent. Cells were then serially trypsinized and passaged three to five times before use in experiments. In preparation for experiments, cells were plated at 3x10^3^-5x10^3^ cells per well in 96 well tissue culture plates and incubated in 1 ml 10% FBS DMEM at 37 °C in 5% CO_2_ for 24–48 h.

### Time-dependent and dose-dependent effects of mitomycin C on corneal fibroblast viability

Normal, GCD II heterozygote, and GCD II homozygote corneal fibroblasts were incubated with 0.02% MMC for 3, 6, and 24 h, after which cell viability was measured using 3-[4,5-demethylthiazol-2,5-diphenyl-2H-tetrazolium bromide] (MTT) and lactate dehydrogenase (LDH) assays. Normal and GCD II homozygote cells were incubated with 0%, 0.01%, 0.02%, and 0.04% MMC for 24 h after which cell viability was measured using MTT and LDH assays. Based on the above findings, subsequent experiments used 0.02% MMC for 6 h. Normal, GCD II heterozygote, and GCD II homozygote cells were incubated with 0.02% MMC for 6 h after which cell viability was measured using LDH assays.

### Lactate dehydrogenase and MTT assays

Cell viability was determined using LDH and MTT assays. The LDH assay (CytoTox 96R Non-Radioactive Cytotoxicity Assay; Promega, Madison, WI) was used to evaluate cellular damage. For the MTT assay, PBS containing 5 mg/ml MTT was added to each well. Plates were incubated for 4 h in the absence of light, the medium was removed, and precipitates were resuspended in 50 µl DMSO. Absorbance at 570 nm or 540 nm was measured using a plate reader. Experiments were performed in triplicate.

### FACS analysis

The number of apoptotic cells was quantified using propidium iodide (PI) staining and flow cytometry. Cells (3x10^5^-5x10^5^ cells) were permeabilized and stained using 5 μl annexin V-FITC and 5 μl propidium iodide (annexin V- FITC Apoptosis Detection Kit; BioVision Inc., Mountain View, CA). DNA staining was analyzed with FACSCalibur (Becton-Dickinson, San Jose, CA).

### Annexin V staining

Cells were stained with annexin V (BioVision Inc.) and then incubated with an avidin-horseradish peroxidase (HRP) complex (1:300). Binding was visualized using a 0.05% diaminobenzidine/0.01% H_2_O_2_ solution. Cells were photographed using a fluorescence microscope equipped with an annexin V filter.

### Reverse transcriptase polymerase chain reaction analysis of *Bcl-xL*, *Bax*, and *TGFBI* mRNA expression

Total cellular RNA was isolated using SV Total RNA Isolation System (Promega) according to the manufacturer’s instructions. The RNA concentration and purity were determined spectrophotometrically (Gene Quant II; Pharmacia Biotech, Cambridge, UK). Total RNA was converted into cDNA using a first-strand synthesis kit (Superscript; Invitrogen-Gibco, Carlsbad, CA).

The PCR reaction for *Bcl-xL* involved 30 cycles of denaturation at 94 °C for 5 min, annealing at 57 °C for 1 min, and extension at 72 °C for 1 min followed by a final incubation at 72 °C for 10 min. The reaction mixture contained primer sequences specific to *Bcl-xL* (5′-GTA AAC TGG GGT CGC ATT GT-3′ and 5′-TGC TGC ATT GTT CCC ATA GA-3′) [22].

The PCR reaction for *Bax* involved 35 cycles of denaturation at 95 °C for 5 min, annealing at 59 °C for 1 min, and extension at 72 °C for 1 min followed by a final incubation at 72 °C for 10 min. The reaction mixture contained primer sequences specific to *Bax* (5′-AGA GGA TGA TTG CCG CCG T-3′ and 5′-CAA CCA CCC TGG TCT TGG ATC-3′) [22].

The reaction mixtures for *TGFBI* were incubated in a thermal controller (Model TPC-100; MJ Research, Watertown, MA) for 27 cycles of denaturation at 94 °C for 5 min, annealing at 55 °C for 45 sec, and extension at 72 °C for 1 min followed by a final incubation at 72 °C for 10 min. The reaction mixture contained primer sequences specific to *TGFBI* (5′-TTG AGA GTG GTA GGG CTG CT-3′ and 5′-GTG TGT GCT GTG CAG AAG GT-3′) and *β-actin* (5′-AGC ACT GTG TTG GCG TAC AG-3′ and 5′-GGA CTT CGA GCA AGA GAT GG-3′).

The quantity of the amplified products was analyzed using an image-documentation system (ImageMaster VDS; Pharmacia Biotech Inc., Uppsala, Sweden). The reverse transcription polymerase chain reaction (RT–PCR) products were analyzed by electrophoresis on 1% agarose gels. *β-actin* levels were used to normalize expression levels for mRNA species assayed. The gels were photographed, and the densities of DNA bands were quantified using a Fluor-S MultiImager (Bio-Rad, Hercules, CA).

### Immunoblot analysis for TGFBIp

Cells were washed with ice-cold PBS and lysed with cell lysis buffer (20 mM HEPES [pH 7.2], 10% glycerol, 10 mM Na_3_VO_4_, 50 mM NaF, 1 mM phenylmethylsulfonyl fluoride [PMSF], 0.1 mM dithiothreitol, 1 µg/ml leupeptin, 1 µg/ml pepstatin, and 1% Triton X-100) on ice for 30 min. The lysate was sonicated, and the homogenates were centrifuged at 15,000x g for 10 min. The protein concentration of the resultant supernatant was determined using the Bradford reagent. Laemmli sample buffer was added to samples of cell lysate protein (15 μg) or culture media (30 μg), which were then boiled for 5 min. The proteins were separated by SDS–PAGE (8% gels) and then transferred to polyvinylidene fluoride (PVDF; Millipore, Billerica, MA) membranes. Membranes were incubated with antibodies against TGFBIp (1:1000 R&D Systems, Inc. Minneapolis, MN) and β-actin (1:10,000 Sigma-Aldrich, St. Louis, MO) followed by HRP-conjugated secondary antibodies. Immunoreactive bands were visualized using an enhanced chemiluminescence kit (ECL kit; Amersham, Piscataway, NJ).

### Statistical analysis

ANOVA was used to compare cell viability. Statistical analysis was performed using SPSS software (version 10.0 for Windows; SPSS Inc., Chicago, IL), and the significance was set at p<0.05.

## Results

### Time- dependent and dose-dependent effects of mitomycin C on corneal fibroblast viability

**Figure 1:**
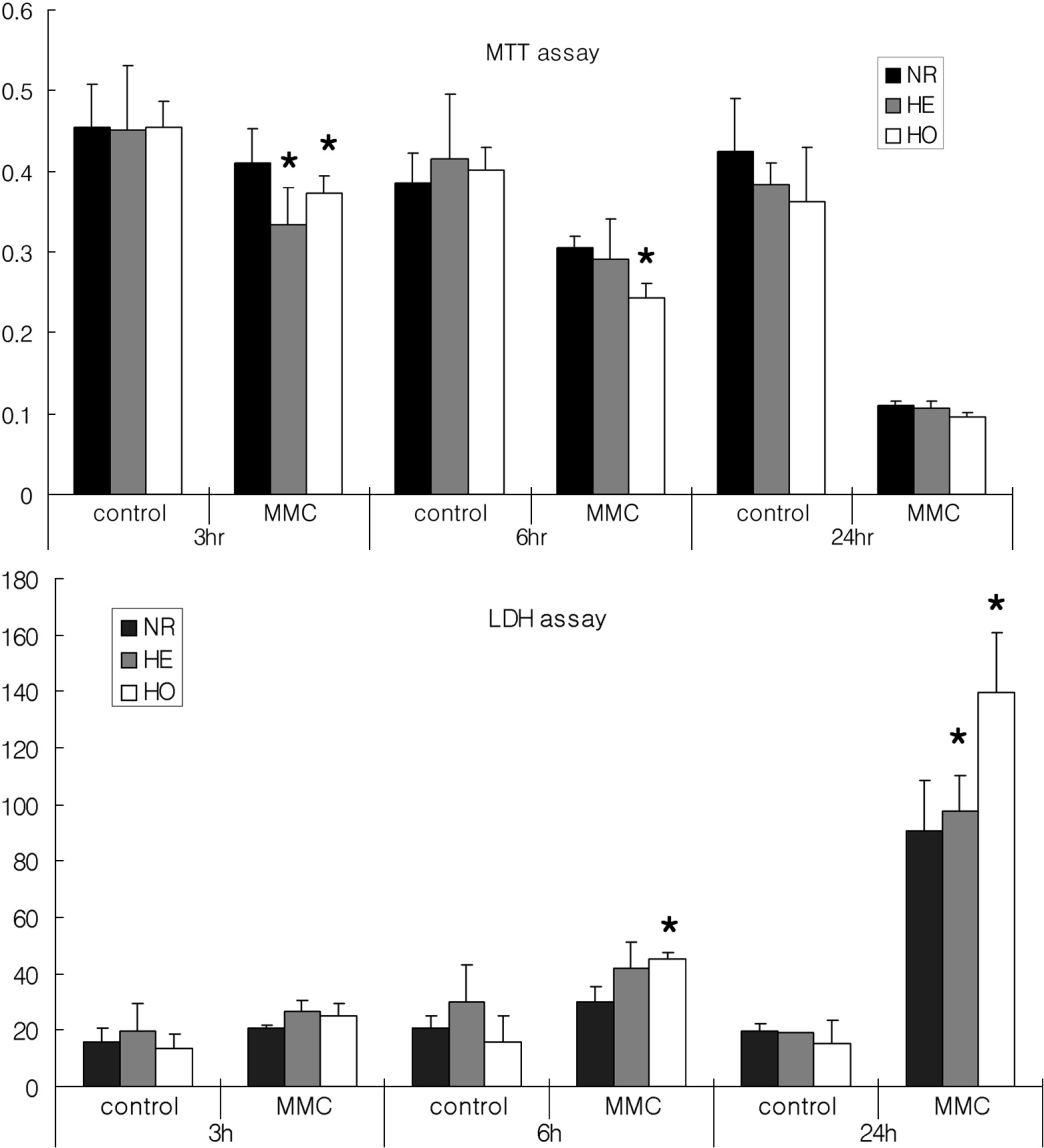
Time course for the effect of 0.02% MMC on corneal fibroblast viability. Corneal fibroblasts were incubated with 0.02% MMC for various times after which cell viability was measured using MTT and LDH assays. The MTT and LDH assays demonstrated some minor differences, there were fewer viable GCD II homozygote cells than normal cells. Upper panel=MTT assay, lower panel=LDH assay. The asterisk indicates a p<0.05; ANOVA. MMC: mitomycin C, control: without MMC, NR: normal, HE: heterozygote, HO: homozygote.

With regard to the time-dependent effects of MMC, there were fewer viable GCD II homozygote cells than normal cells at all time points (p=0.043 at 3 h, p=0.023 at 6 h, and p=0.350 at 24 h for MTT assay; p=0.086 at 3 h, p=0.043 at 6 h, and p<0.01 at 24 h for LDH assay; Figure 1). While the MTT and LDH assays demonstrated some minor differences, there were fewer viable GCD II homozygote cells than normal cells at 6 h.

**Figure 2:**
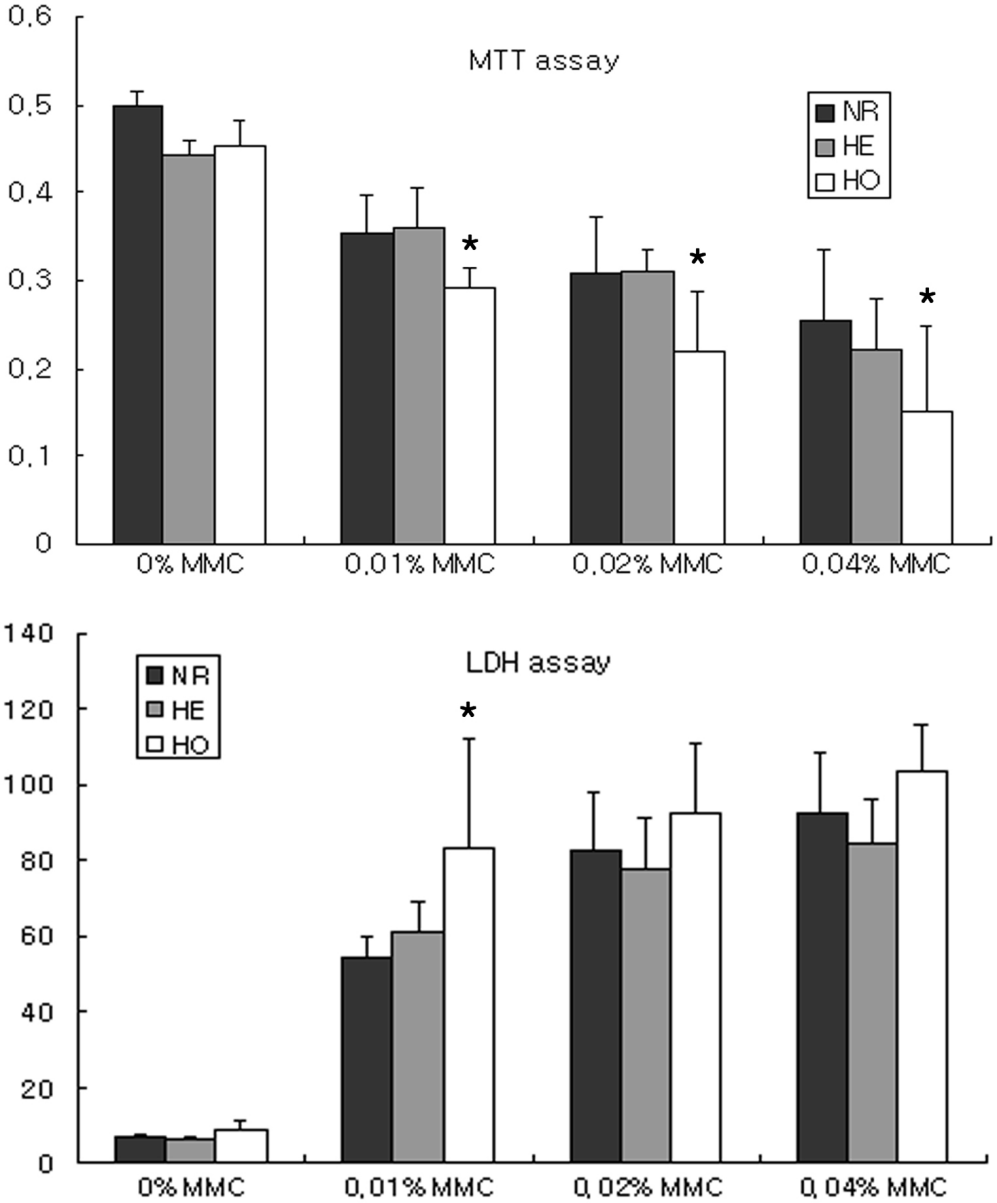
Concentration-dependent effect of MMC on corneal fibroblast viability. Cells were incubated with various concentrations of MMC for 24 h after which cell viability was measured using MTT and LDH assays. The MTT assay demonstrated fewer viable GCD II homozygote cells than normal cells in all concentrations. Upper panel=MTT assay, lower panel=LDH assay. The asterisk indicates a p<0.05; ANOVA. MMC: mitomycin C, control: without MMC, NR: normal, HE: heterozygote, HO: homozygote.

**Figure 3:**
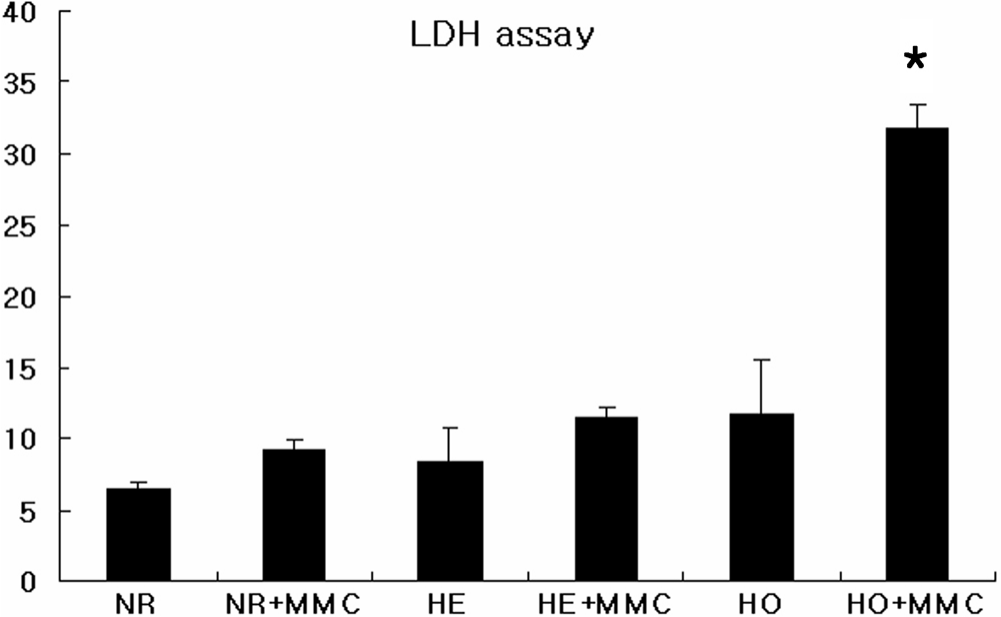
The effect of 0.02% MMC for 6 h on corneal fibroblast viability. Cells were incubated with 0.02% MMC for 6 h after which cell viability was measured using LDH assays. There were fewer viable GCD II homozygote cells, compared to other cell types. The asterisk indicates a p<0.05; ANOVA. MMC: mitomycin C, NR: normal, HE: heterozygote, HO: homozygote.

In regard to the dose-dependent effects of MMC, there was a reduction in the number of viable GCD II homozygote cells at all concentrations by MTT assay (p=0.028 at 3 h, p<0.01 at 6 h, and p<0.01 at 24 h for MTT assay; p=0.034 at 3 h, p=0.065 at 6 h, and p<0.125 at 24 h for LDH assay; Figure 2). In the LDH assay, there were fewer viable GCD II homozygote cells at 0.01% compared to normal cells. After incubation with 0.02% MMC for 6 h, there were fewer viable GCD II homozygote cells compared to other cell types (Figure 3).

### Mitomycin C induces apoptosis

**Figure 4:**
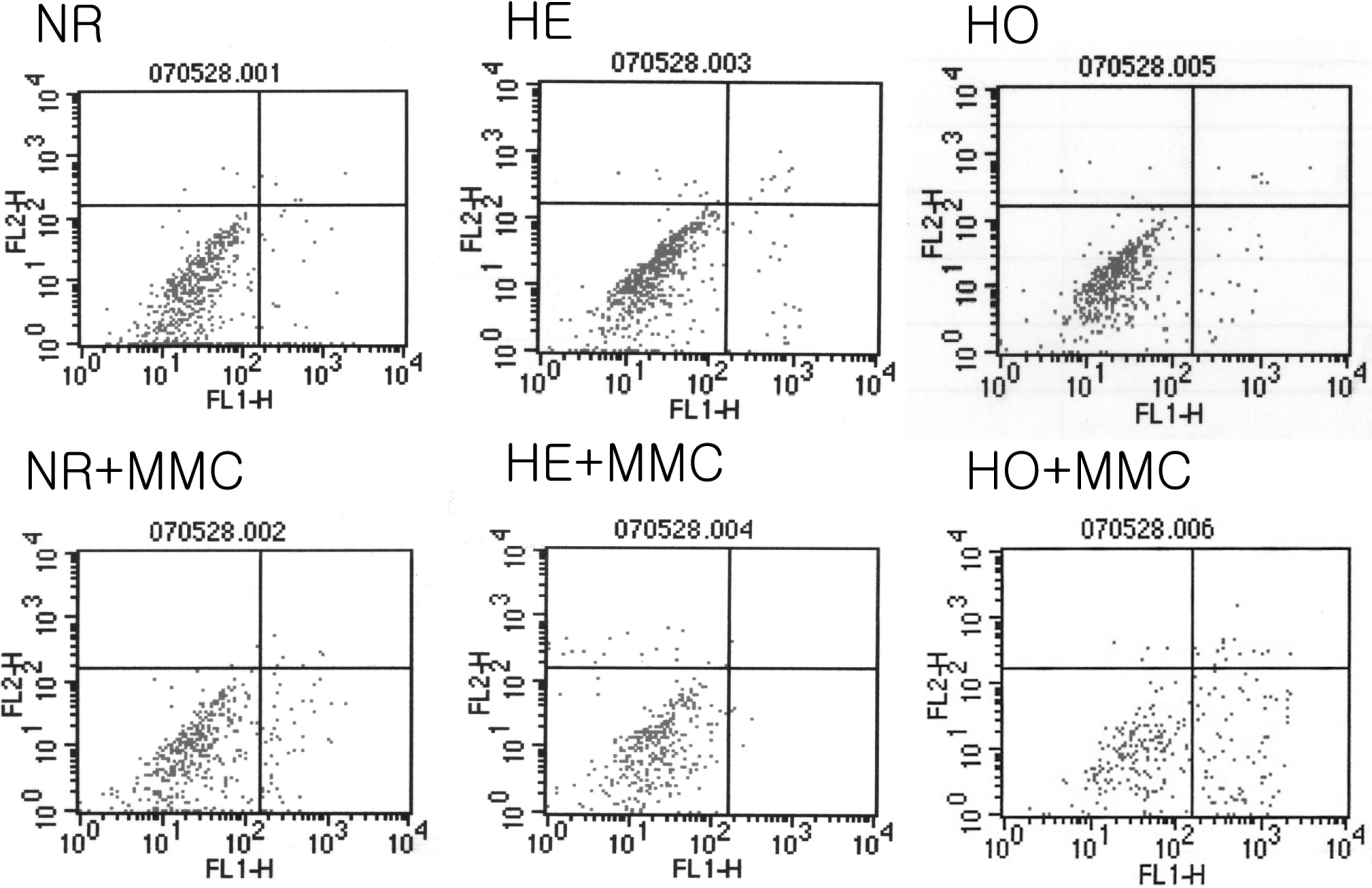
The effect of MMC on corneal fibroblast apoptosis: FACS analysis. Cells were incubated with 0.02% MMC for 6 h after which they were harvested, the DNA was stained with propidium iodide, and the cells were analyzed using FACS. MMC increased the proportion of apoptotic cells in GCD II homozygote cells. MMC: mitomycin C, NR: normal, HE: heterozygote, HO: homozygote.

Cells were incubated with 0.02% MMC for 6 h after which they were stained and underwent FACS analysis to determine the amount of apoptosis and necrosis. MMC increased the proportion of apoptotic cells particularly in GCD II homozygote cells (Figure 4).

**Figure 5:**
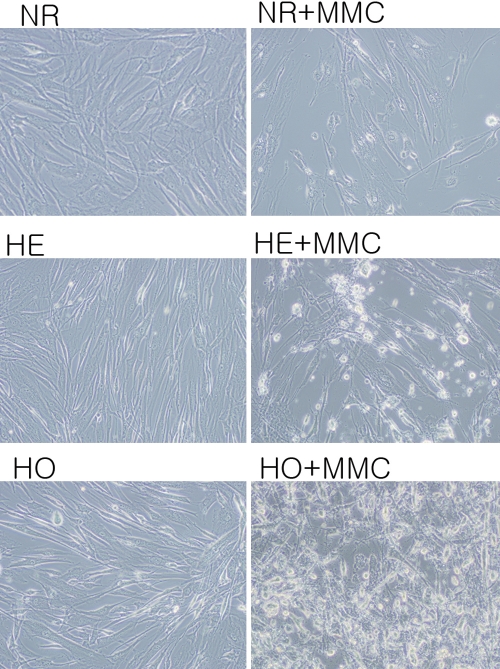
The effect of MMC on corneal fibroblast apoptosis. Cells were incubated with 0.02% MMC for 6 h after which they were stained with annexin V then avidin-horseradish peroxidase (HRP) complex (1:300), and binding was visualized using a 0.05% diaminobenzidine/0.01% H_2_O_2_ solution. Cells were photographed using a fluorescence microscope equipped with an annexin V filter. Following MMC incubation, the GCD II homozygote cells showed cytoplasmic shrinkage and perimembranous stippling prominently compared with normal cells. MMC: mitomycin C, NR: normal, HE: heterozygote, HO: homozygote.

Cells were incubated with 0.02% MMC for 6 h after which they were stained for annexin V to find apoptotic changes. Cells undergoing apoptosis experienced cytoplasmic shrinkage and perimembranous stippling. Following MMC incubation, the GCD II homozygote cells strongly stained positive for annexin V and displayed typical characteristics of apoptosis while such staining was less apparent in normal cells (Figure 5).

### Expression of *Bcl-xL*, *Bax*, and *TGFBI*

**Figure 6:**
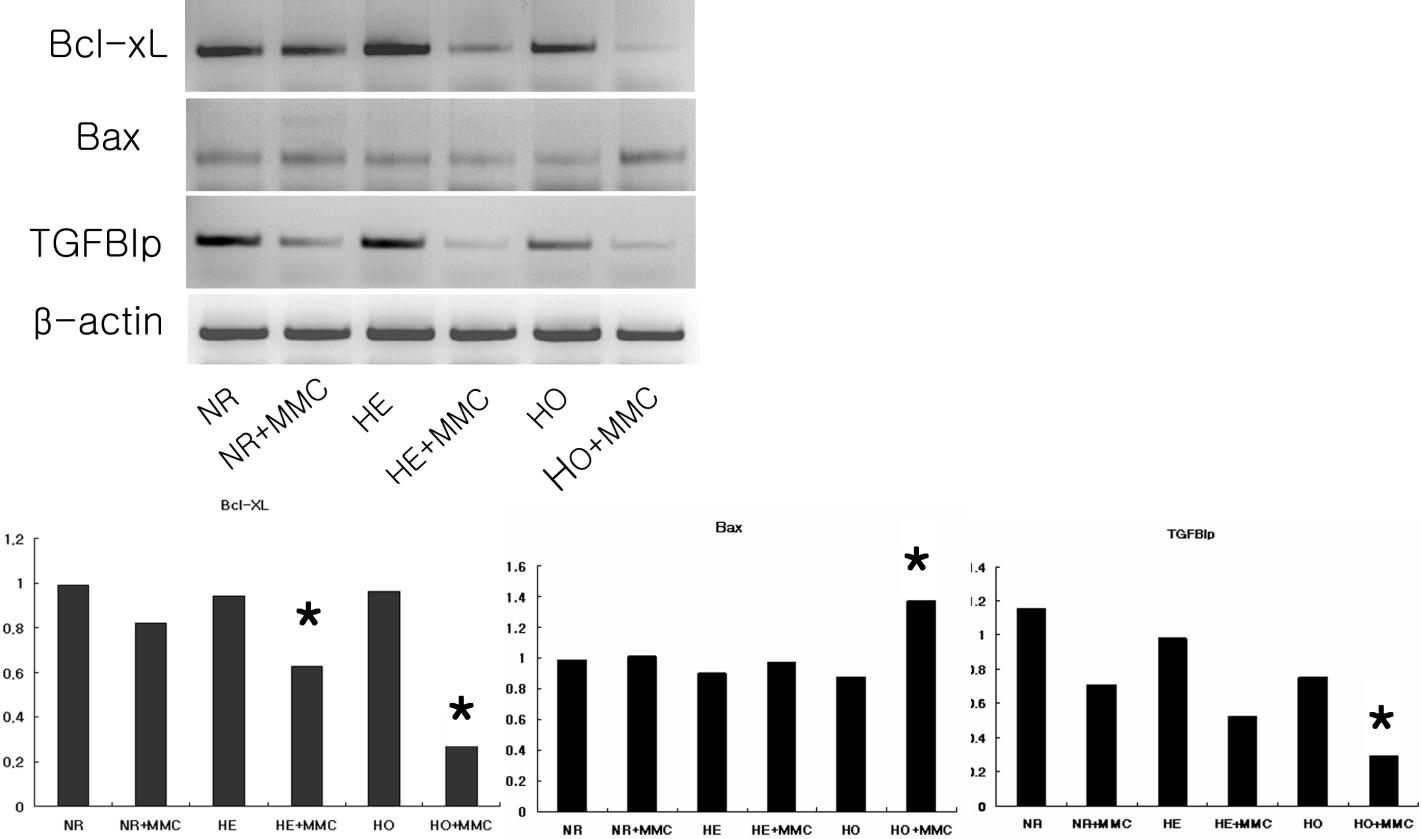
The effect of MMC on *Bcl-xL*, *Bax*, and *TGFBI* mRNA expression in corneal fibroblasts. Cells were incubated with 0.02% MMC for 6 h after which RNA was isolated and subjected to RT–PCR analysis to determine the levels of *Bcl-xL*, *Bax*, and *TGFBI* mRNA expression. MMC reduced Bcl-xL mRNA expression in heterozygote (p=0.045) and homozygote (p=0.017) cells and markedly enhanced Bax expression in GCD II homozygote cells (p<0.01). MMC decreased *TGFBI* mRNA expression statistically significant in homozygote cells (p=0.025). The asterisk indicates a p<0.05; ANOVA compared with normal cell. MMC: mitomycin C, NR: normal, HE: heterozygote, HO: homozygote.

Cells were incubated with 0.02% MMC for 6 h after which they were harvested, RNA was isolated, and RT–PCR analysis was performed to determine the expression levels of *TGFBI* and the apoptosis-related factors, *Bcl-xL* and *Bax*. MMC reduced *Bcl-xL* mRNA expression in heterozygote (p=0.045) and homozygote (p=0.017) cells and markedly enhanced *Bax* expression in GCD II homozygote cells (p<0.01). Before the application of MMC, corneal fibroblasts expressed *TGFBI* mRNA at similar levels. However, MMC treatment decreased *TGFBI* mRNA expression in all three cell types. The results were statistically significant for homozygote cells (p=0.025; Figure 6).

**Figure 7:**
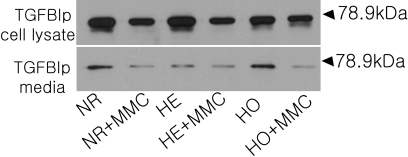
The effect of MMC on TGFBIp protein expression. Cells were incubated with 0.02% MMC for 6 h after which samples of media were taken, and the cells harvested. Cell lysates (15 μg) and media (30 μg) were analyzed for TGFBIp protein levels using immunoblotting. MMC reduced TGFBIp levels in both cell lysates and media for all 3 cell types. MMC: mitomycin C, NR: normal, HE: heterozygote, HO: homozygote.

Cells were incubated with 0.02% MMC for 6 h after which samples of media were taken and the cells were harvested. Cell lysates and media were analyzed for TGFBIp protein levels via immunoblotting. MMC reduced TGFBIp levels in both cell lysates and media for all three cell types (Figure 7).

## Discussion

MMC was first introduced as an agent that could modulate corneal wound healing processes and reduce corneal haze after refractive procedures [23]. Subsequently, MMC was used to treat recurrent subepithelial fibrosis after refractive procedures, radial keratotomy (RK), and PRK or for management of complications such as ‘button hole’ [24]. MMC has even been applied during the LASEK procedure in normal corneas to improve vision after surgery [25]. However, the antiproliferative effect of this drug on keratocytes has led investigators to fear possible long-term keratocyte depletion and delayed wound healing [26]. 

We have reported that the intraoperative use of topical 0.02% MMC with PTK prevented or delayed the recurrence of GCD II [18]. Subsequently, topical MMC was effective in preventing the recurrence of Reis-Bücklers dystrophy after PTK [27]. MMC has also been considered for reducing side effects related to excessive wound healing or recurrence of corneal dystrophy after corneal surgery [18,27,28]. Despite the widespread clinical use of MMC, there are few in vitro studies that have supported such use or few that have provided information regarding its mechanism of action.

The present study examined the effect of MMC on viability in normal, GCD II heterozygote, and GCD II homozygote corneal fibroblasts. The study also sought to determine the molecular mechanisms underlying MMC activity. MMC reduced cell viability in a time-dependent and dose-dependent manner in all three cell types. However, GCD II homozygote cells were the most susceptible. FACS analysis and annexin V staining showed that apoptosis was the cause of cell death in all three cell types. The present data are consistent with a previous study showing that MMC induces keratocyte apoptosis in a time-dependent and dose-dependent manner [29]. Also, the ratio between apoptotic and live cells was clearly highest in GCD II homozygote cells.

The current findings suggest that GCD II homozygote keratocytes are more susceptible to MMC-induced apoptosis than normal keratocytes and that the application of MMC during surgery would have a greater effect on GCD II homozygote corneas than on normal ones. Previous studies showed that MMC-induced keratocyte apoptosis in normal corneas reduced the number of cells and delayed epithelial healing but did not significantly disturb corneal anatomic structure [30]. The decreased number and activity of keratocytes appears to reduce corneal haze and fibrosis during wound healing in the cornea [31]. In the present study, all three cell types had similar levels of baseline apoptosis before the addition of MMC. Different levels of apoptosis were observed in each cell type only after MMC treatment.

Bcl-xL proteins are inhibitors of the mitochondrial apoptosis pathway. Bcl-xL proteins block the activity of their proapoptotic counterparts including Bax, thereby preventing the release of cytochrome c and the activation of caspase [32,33]. Furthermore, Bcl-xL is capable of preventing cell death, suggesting that these proteins exert independent effects on the mitochondrial apoptotic pathway. In the present study, the addition of MMC resulted in decreased *Bcl-xL* mRNA expression in all cell types. In addition, MMC cause a marked increase in *Bax* mRNA expression in GCD II homozygote cells. MMC-induced corneal fibroblast apoptosis is associated with the mitochondrial pathway and release of cytochrome c [29]. These findings indicate that GCD II homozygote cells were less able to resist MMC-induced triggering of the mitochondrial apoptotic pathway.

The main component of corneal deposits in GCD II patients is considered to be the mutant TGFBIp. Therefore, *TGFBI* mRNA and secreted protein levels may influence the severity of corneal deposits. Whether epithelial cells or keratocytes are the main source of TGFBIp in TGF-β1-induced corneal dystrophies is still under debate. Evidence of the former includes the location of the deposits in granular corneal dystrophy and of the initial graft recurrences in the subepithelial region following penetrating keratoplasty (PK) as well as the fact that the stroma is only affected later [34]. However, the present study showed that corneal fibroblasts express *TGFBI* mRNA and produce TGFBIp protein that is secreted into the media. MMC caused similar relative reductions in *TGFBI* mRNA and protein levels across all three cell types. Using in situ hybridization, a previous report showed that *TGFBI* mRNA was expressed not only in the epithelial layer but also in the stromal layer during the wound healing process [35]. Therefore, in combination with PTK, the inhibitory effect of MMC on *TGFBI* mRNA expression in corneal fibroblasts would be a dual acting treatment for GCD II with PTK removing pre-existing deposits and MMC suppressing the accumulation of new deposits. However, these effects were observed only in cell culture. Therefore, possible differences of cell responses to MMC between cell culture and living cornea should be considered before being used on patients. To validate this dual effect, the clinical application of PTK with MMC in corneal dystrophy patients and long-term follow-up are required.

The present study found that MMC caused similar but more extensive changes in mutated GCD II corneal fibroblasts compared to normal cells such as the induction of apoptosis and reduced synthesis of TGFBIp. The findings may explain the MMC-dependent suppression or delay of recurrence in GCD II corneas after PTK. Our previous study suggested that this treatment only delayed recurrence in homozygote GCD II patients. The effects of MMC in the present study were only partial and did not completely suppress the production of TGFBIp, which may explain the limited effect of MMC in severe conditions such as homozygote corneas.
